# Hydrogen-rich saline alleviates early brain injury through regulating of ER stress and autophagy after experimental subarachnoid hemorrhage

**DOI:** 10.1590/ACB360804

**Published:** 2021-10-08

**Authors:** Bingjie Jiang, Yunping Li, Weimin Dai, An Wu, Huayong Wu, Dandan Mao

**Affiliations:** 1 MM. Department of Neurosurgery - The Quzhou Affiliated Hospital - Wenzhou Medical University - Quzhou People’s Hospital - Quzhou, China.; 2 MM. Department of Neurosurgery - The Quzhou Affiliated Hospital - Wenzhou Medical University - Quzhou People’s Hospital - Quzhou, China.; 3 BS. Department of Neurosurgery - The Quzhou Affiliated Hospital - Wenzhou Medical University - Quzhou People’s Hospital - Quzhou, China.; 4 MM. Department of Neurosurgery - The Quzhou Affiliated Hospital - Wenzhou Medical University - Quzhou People’s Hospital - Quzhou, China.; 5 MM. Department of Neurosurgery - The Quzhou Affiliated Hospital - Wenzhou Medical University - Quzhou People’s Hospital - Quzhou, China.; 6 MM. Department of Neurosurgery - The Quzhou Affiliated Hospital - Wenzhou Medical University - Quzhou People’s Hospital - Quzhou, China.

**Keywords:** Hydrogen, Brain Injuries, Oxidative Stress, Reactive Oxygen Species, Autophagy

## Abstract

**Purpose:**

Subarachnoid hemorrhage (SAH) is a common complication of cerebral vascular disease. Hydrogen has been reported to alleviate early brain injury (EBI) through oxidative stress injury, reactive oxygen species (ROS), and autophagy. Autophagy is a programmed cell death mechanism that plays a vital role in neuronal cell death after SAH. However, the precise role of autophagy in hydrogen-mediated neuroprotection following SAH has not been confirmed.

**Methods:**

In the present study, the objective was to investigate the neuroprotective effects and potential molecular mechanisms of hydrogen-rich saline in SAH-induced EBI by regulating neural autophagy in the C57BL/6 mice model. Mortality, neurological score, brain water content, ROS, malondialdehyde (MDA), and neuronal death were evaluated.

**Results:**

The results show that hydrogen-rich saline treatment markedly increased the survival rate and neurological score, increased neuron survival, downregulated the autophagy protein expression of Beclin-1 and LC3, and endoplasmic reticulum (ER) stress. That indicates that hydrogen-rich saline-mediated inhibition of autophagy and ER stress ameliorate neuronal death after SAH. The neuroprotective capacity of hydrogen-rich saline is partly dependent on the ROS/Nrf2/heme oxygenase-1 (HO-1) signaling pathway.

**Conclusions:**

The results of this study demonstrate that hydrogen-rich saline improves neurological outcomes in mice and reduces neuronal death by protecting against neural autophagy and ER stress.

## Introduction

Subarachnoid hemorrhage (SAH) is a common complication of cerebral vascular disease that is associated with a high rate of mortality, morbidity, and poor prognosis, especially in patients with hypertension. An occurrence of 6.2-10 per 100,000 has been recorded in Western countries^1^^-^ 3. The key causes for SAH patients’ poor outcomes were early brain damage (EBI) and cerebral vasospasm (CVS)^4^. Recent clinical trials, however, have shown that drugs can greatly reduce CVS while having little impact on outcomes following SAH^5^, and previous clinical studies demonstrated it too^6^. The latest research has shown that EBI after SAH appears to play a critical role^7^^-^ 10. The possible mechanisms underlying EBI include autophagy, apoptosis, direct neuronal death, and necroptosis^8^^,^ 11 ^-^ 13. However, Zille^14^ reported that inhibitors of caspase-dependent apoptosis, protein or mRNA synthesis, autophagy, mitophagy, or parthanatos had no effect in vitro or in vivo after intracerebral hemorrhage (ICH). Instead, inhibitors of ferroptosis defended against toxicity caused by hemoglobin and hemin. To date, it is unknown how often various types of cell death play a role in SAH-induced toxicity.

Under various physiological and pathological settings, autophagy is the primary cellular lysosomal degradation process for degrading and recycling intracellular proteins and organelles^15^. Autophagy has been shown to play a critical function in many central nervous system disorders, including traumatic brain injury (TBI)^16^^-^ 18, ICH^19^, SAH^8^, and Huntington’s disease^20^. Tang^17^ found that inhibiting autophagy greatly reduce neuronal apoptosis and necrotic cell death, but the autophagy activator rapamycin can exacerbate brain injury. In turn, Fang^18^ stated that activating autophagy can reduce mitochondrial apoptosis, boost neurological function, cerebral edema, and relieve blood-brain barrier (BBB) disturbance after TBI in mice. Until nowadays, it was uncertain whether autophagy’s neuroprotection was dependent on stimulation or inhibition. It was beneficial to investigate new possible drug targets focused on autophagy. Endoplasmic reticulum (ER) is the largest cellular organelle, in which all secreted and membrane proteins are synthesized and properly folded^21^. Previous studies had confirmed that ER stress play a vital important role in the early brain injury after SAH^21^^-^ 23.

Recently, hydrogen gas or hydrogen-rich saline have been commonly recognized for their ability to defend against a variety of diseases, including ischemia-reperfusion damage, stroke, spontaneous subarachnoid hemorrhage (SAH), and neurodegenerative diseases, by controlling oxidative stress, inflammatory response, and neuronal apoptosis^24^^-^ 27. Hydrogen has been shown in several experiments to selectively suppress hydroxyl radicals and peroxynitrites, and hence plays an important role in antioxidant, anti-apoptotic, anti-inflammatory, and cytoprotective properties^24^^-^ 28. However, the neuroprotective effects of hydrogen-rich saline therapy on SAH are debatable. Heme oxygenase-1 (HO-1) is a cellular resistance enzyme that is caused by and protects against oxidant-induced damage. In the central nervous system, HO-1 has anti-necroptosis, anti-neuroinflammatory, and neuroprotective effects (central nervous system – CNS)^28^^,^ 29. Previous study also confirmed that Nrf2/HO-1 can regulate neuron death in acute CNS disease^30^. Thus, therapies targeting Nrf2 and HO-1 may be potential treatments for protection against inflammation, oxidative stress, and necroptosis after SAH. However, the exact mechanisms of the neuroprotective effects of hydrogen-rich saline therapy remain unclear. It was investigated here the neuroprotective effect of hydrogen-rich saline therapy in a mice model of SAH through effects on neuroinflammation and necroptosis, and whether the neuroprotection was dependent on the ROS/Nrf2/HO-1 pathway.

## Methods

The study protocol was approved by the Quzhou Affiliated Hospital of Wenzhou Medical University Research Ethics Committee (WYLL-2020-11). All animal experiments performed for this study complied with the National Institutes of Health guidelines for the handling of laboratory animals and were approved by the Ethics Committee of the Wenzhou Medical University. All experiments were conducted on healthy adult male C57BL/6J mice(8-10 weeks, 22-25 g) (Wenzhou Medical University, Wenzhou, China). Twenty-four mice were set in each group. The mice were housed in animal care facilities with a 12 h light/dark cycle and had free access to food and water.

### Animals SAH model

The endovascular perforation method was used to construct the SAH model based on a protocol that was previously described^7^^,^ 31. Briefly, male C57BL6/J mice were anesthetized by intraperitoneal (i.p.) injection of 50-mg/kg pentobarbital sodium. Rectal temperature was kept at 37 ± 0.5°C during operation using a heating pad. A midline incision was made in the neck, and left common external and internal carotid arteries were exposed. The left external carotid artery was ligated and cut, leaving a 3-mm stump, and a 4–0 (0.33 mm) monofilament nylon suture, 15 mm in length, was inserted into the left internal carotid artery through the external carotid artery stump to perforate the artery at the bifurcation of the anterior and middle cerebral artery. The suture was advanced 3 mm further to perforate the bifurcation of the anterior and middle cerebral arteries. After approximately 10 s, the suture was withdrawn. Sham rats received similar surgical procedures, but without perforation.

### Drug preparation and administration

After the SAH model was established successfully, animals were given daily intraperitoneal injections of either hydrogen-rich (5 mL/kg) (experimental) or plain (control) saline for 72 hours. The preparation of hydrogen-rich was according to the previous study^32^^,^ 33. Briefly, purified H_2_ was dissolved in normal saline for 2 hours under high pressure with 0.4 MPa, and the physiological concentration was kept at 1.73 mL hydrogen per 100 mL saline (average, more than 6 mmol/L). Hydrogen-rich saline was stored at 4°C in an aluminum bag with no dead volume under atmospheric pressure. Hydrogen-rich saline was freshly prepared every week to ensure a constant concentration. The content of hydrogen in saline was evaluated and detected by gas chromatography, as a previous study reported^34^.

### Neurological function assessment

The severity of early brain injury was evaluated by neurological function at 48 hours after SAH using a previously described neurological grading system^7^. The scoring system consisted of six tests, and specific standards are shown in Supplementary Table 1. The neurological score, ranged from 3 to 18, included spontaneous activities (0-3), movement symmetry of all limbs (0-3), forelimbs outstretching (0-3), body proprioception (1-3), response to vibrissae touch (1-3) and climbing (1-3). All rats from every group received a behavioral assessment, and a higher score represented a better neurological function.

### Mortality and SAH grade

Mortality was documented 48 hours after SAH. SAH grade was given according to a previously described grading system^35^. Briefly, the grading was given based on subarachnoid blood blot:

- grade 0: no subarachnoid blood;
- grade 1: minimal subarachnoid blood;
- grade 2: moderate blood clot with appreciable arteries;
- grade 3: blood clot obliterating all arteries within the segment.

The grade ranges from 0 to 18. Mice with SAH grading scores of less than 7, which had no prominent brain injury, were excluded from the study.

### Brain water content

The severity of brain edema was evaluated by brain water content, which was determined by the standard wet-dry method as in previous studies^7^^-^ 10. The rats were sacrificed 48 hours after SAH, and the entire brain was harvested and separated into the left and right cerebral hemispheres, followed by weighting cerebellum and brain stem (wet weight). Then, brain specimens from each group were dehydrated at 105°C for 24 hours to acquire the dry weight. The percentage of brain water content was equal to (wet weight - dry weight)/wet weight × 100%.

### Evans blue extravasation

Evans blue extravasation was performed as previously described^36^. Briefly, mice were anesthetized by pentobarbital sodium (50 mg/kg) injection 48 hours after ICH/obstructive sleep apnea (OSA). Evans blue dye (2%, 5 mL/kg; Sigma-Aldrich, St. Louis, MO, United States) was injected into the left femoral vein over 2 min and circulated for 60 min. Then, the mice were sacrificed with 100 mg/kg sodium pentobarbital via intraperitoneal injection and phosphate-buffered saline (PBS) intracardial perfusion. Death was clarified by observing respiration and by using the corneal reflection method. The brains were removed and quickly divided into the left and right cerebral hemispheres, weighed, homogenized in saline, and centrifuged at 15,000 g for 30 min. Subsequently, the resultant supernatant was added with an equal volume of trichloroacetic acid, incubated overnight at 4°C, and centrifuged at 15,000 g for 30 min. Next, the resultant supernatant was collected and spectrophotometrically quantified at 610 nm for Evans blue dye.

### Analysis of reactive oxygen species

The non-fluorescent diacetylated 2′,7′-dichlorofluorescein (DCF-DA) probe (Sigma-Aldrich, St. Louis, MO, United States), which becomes highly fluorescent upon oxidation, was used to evaluate intracellular ROS production according to the manufacturer’s instructions^37^.

### Analysis of lipid peroxidation

Malondialdehyde (MDA) levels were detected by lipid peroxidation assay kit (Ex/Em 532/553 nm, Ab118970, Abcam, Cambridge, United Kingdom), according to the manufacturer’s instructions^38^.

### TUNEL staining

A terminal deoxynucleotidyl transferase dUTP nick end labeling (TUNEL) assay was conducted to assess neuronal death in the brain cortex according to the previous study^31^. TUNEL reaction mixture (50 μL) was added to each sample, and the slides were incubated in a humidified dark chamber for 60 min at 37°C. The slides were then incubated with DAPI for 5 minutes at room temperature in the dark to stain the nuclei, followed by imaging with a fluorescence microscope. The procedure was performed according to the manufacturer’s instructions with a TUNEL staining kit. A negative control (without the TUNEL reaction mixture) was used. The apoptotic index (%) was the ratio of the number of TUNEL-positive cells/total number of cells × 100. The cell count was confirmed in four randomly selected high-power fields, and the data obtained from each field were averaged.

### Western blot analysis

Western blot analysis was performed as indicated previously^39^. Briefly, cerebral cortex or hippocampus samples were collected, dissolved, and separated by sodium dodecyl sulfate-polyacrylamide gel electrophoresis in 10% polyacrylamide gels. A BCA protein assay kit (Beyotime) was used to measure protein concentrations by the bicinchoninic acid method. Then, protein samples were transferred onto immobilon nitrocellulose membranes. The membranes were blocked at room temperature for 1 h with 5% nonfat milk.

The membranes were then incubated with the following primary antibodies overnight at 4°C:

- rabbit anti-β-actin (1:1,000, Abcam, ab8227);
- rabbit CHOP (#5554, Cell Signaling, 1:1,000);
- rabbit anti-cleaved-caspase-12 (#2202, Cell Signaling, 1:200);
- rabbit anti-GRP78 (#3183, Cell Signaling, 1:800);
- rabbit anti-Beclin-1 (1 μg/mL, Abcam, ab62557);
- rabbit anti-Nrf2 (1:1,000, rabbit polyclonal, Abcam, ab31163);
- rabbit anti-HO-1 (1:1,000, rabbit polyclonal, Abcam, ab13243);
- rabbit anti-LC-3B (1 μg/mL, rabbit monoclonal, Abcam, ab48394).

After washing the membranes with TBST three times, HRP-conjugated goat anti-rabbit IgG or goat anti-mouse IgG secondary antibodies (1:5,000) were applied, and the membranes were incubated in the secondary antibodies at room temperature for 1.5 h. The protein bands were detected using a Bio-Rad imaging system (Bio-Rad, Hercules, CA, United States) and quantified with ImageJ.

### Statistical analysis

All experiments were repeated more than three times, and the data are expressed as the means and scanning electron microscope (SEM). Statistical Package for the Social Sciences 14.0 (SPSS, Chicago, IL, United States) and GraphPad Prism 6 (GraphPad Software, San Diego, CA, United States) were used for the statistical analyses. Student’s t-test was used when two groups were compared, and one-way analysis of variance (ANOVA) followed by Bonferroni’s post-hoc test was used for the comparison of two independent variables. For non-normally distributed data and/or non-homogeneous variance, Kruskal-Wallis test was used followed by Dunn’s post-hoc test. For all the statistical analyses, data were considered significant at p < 0.05.

## Results

### Treatment with hydrogen-rich saline has no long-term effects neither on mortality rates nor on SAH grade in SAH models

**Figure 1:**
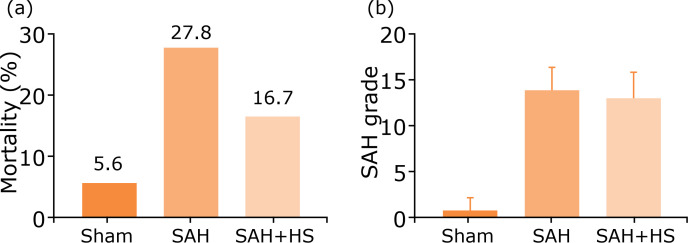
Treatment with hydrogen-rich saline has no long-term effects neither on mortality rates nor on SAH grade in SAH models. **(a)** Mortality rates in the sham group (5.6%), SAH group (27.8%), and the SAH + HS group (16.7%). No significant differences between the three groups. **(b)** SAH grade scores in the sham group, the SAH group, and the SAH + HS group, which showed no significant differences (one-way analysis of variance [ANOVA]).

SAH: Subarachnoid hemorrhage; HS: hydrogen-rich saline.

To clarify the neuroprotection of hydrogen-rich saline, the endovascular perforation method was used to construct the SAH model in vivo. The effect of hydrogen-rich saline treatment on the neurological damage parameters was evaluated, including mortality rates and SAH grades. As shown in Fig. 1, mortality rates (Fig. 1a) and SAH grades (Fig. 1b) in various groups, including sham, SAH, SAH+ hydrogen-rich saline (SAH+HS) did not significantly differ, suggesting that hydrogen-rich saline treatment has no effects in alleviating SAH in long term. So, the focus was on assessing the value of hydrogen-rich saline treatment on early brain injury in the following studies.

### Hydrogen-rich saline alleviates EBI after SAH

To clarify the neuroprotection of hydrogen-rich saline after SAH, modified neurological severity scores were used to evaluate neurological deficits, and brain water content by the wet-dry and Evans blue extravasation method at 48 h after SAH to evaluate brain damage.

**Figure 2:**
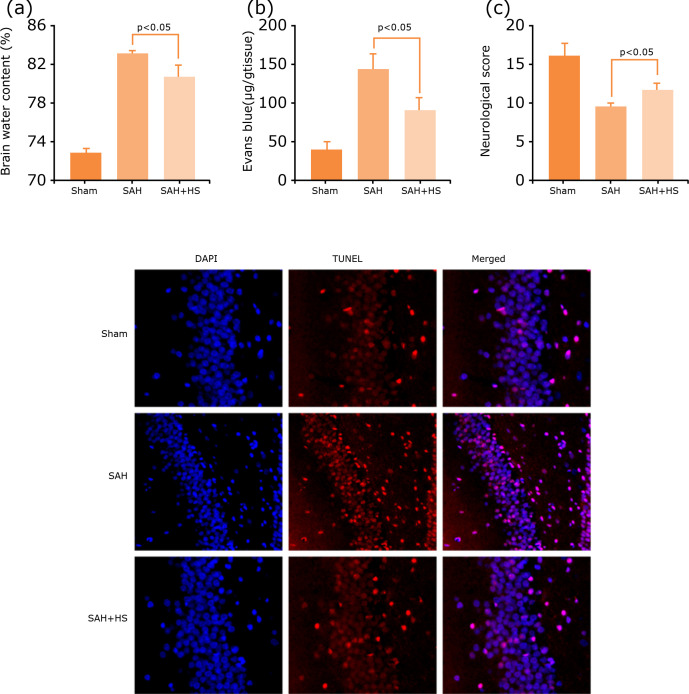
Hydrogen-rich saline alleviates EBI after SAH. **(a)** Hydrogen-rich saline alleviates brain water content significantly after SAH (n=6, p<0.05). **(b)** Hydrogen-rich saline alleviates BBB permeability after SAH (n=6, p<0.05). **(c)** Neurological score of mice in the sham group, SAH group and SAH+HS group at 48 h, hydrogen-rich saline increased the neurological score significantly (n=10, p<0.05). **(d)** TUNEL assay showed that hydrogen-rich saline alleviates neuronal death. p<0.05; ANOVA; mean ± SEM.

SAH: subarachnoid hemorrhage; HS: hydrogen-rich saline; TUNEL: terminal deoxynucleotidyl transferase dUTP nick end labeling; EBI: early brain injury; BBB: blood-brain barrier; ANOVA: analysis of variance; SEM: scanning electron microscope.

The results showed that SAH increased the brain water content significantly (p<0.05, Fig. 2a), and BBB permeability (p<0.05, Fig. 2b), which was alleviated after hydrogen-rich saline treatment. Similar results were found in neurological scores, which were decreased significantly after SAH, and hydrogen-rich saline induction can significantly improve the neurological function (p<0.05, Fig. 2c). Neuronal damage and death were the main reason that leads to EBI after SAH. So, TUNEL assay was used to evaluate the level of cell death in treated and untreated with hydrogen-rich saline in the SAH mice at 48 h after model construction. The hippocampus neuronal death decreased after hydrogen-rich saline treatment (Fig. 2d). These results demonstrate that hydrogen-rich saline has neuroprotective effects after SAH.

### Hydrogen-rich saline inhibited SAH-induced autophagy activation in the hippocampus

**Figure 3:**
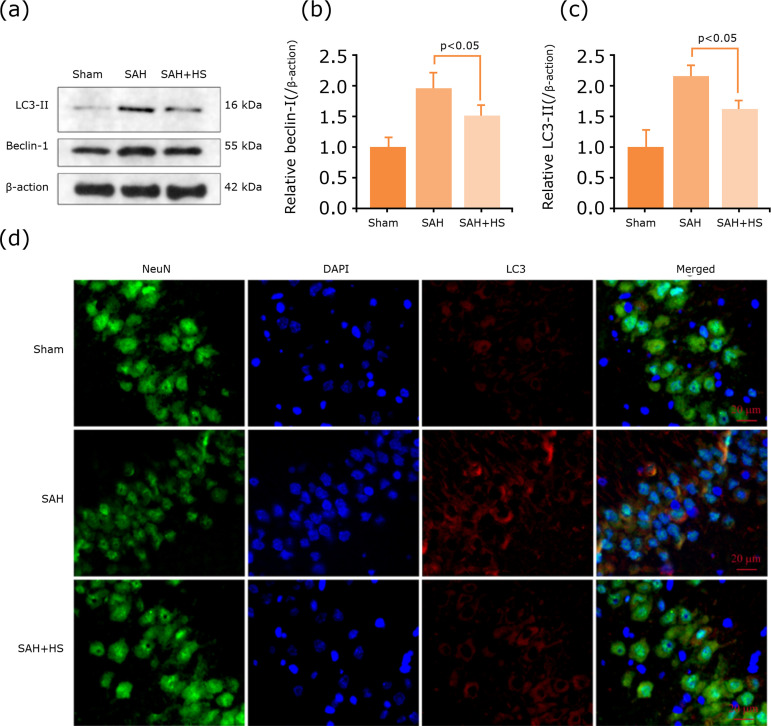
Hydrogen-rich saline inhibited SAH-induced autophagy activation in the hippocampus. **(a)** Expression of autophagy-related proteins, Beclin-1 and LC3 in the hippocampus of mice after SAH were determined by Western blotting. **(b-c)** Quantification of Beclin-1 and LC3 protein levels in the hippocampus to actin loading control, hydrogen-rich saline decreased Beclin-1 and LC3 expression after SAH in mice. **(d)** Immunocytochemistry shows that hydrogen-rich saline downregulated LC3 expression in the hippocampus. n=6; p<0.05; ANOVA; mean ± SEM.

SAH: subarachnoid hemorrhage; HS: hydrogen-rich saline; ANOVA: analysis of variance; SEM: scanning electron microscope.

To clarify whether autophagy plays an important role in SAH and hydrogen-rich saline can regulate autophagy, the expression levels of autophagy-related protein by western blotting were also detected (Fig. 3a). The results of western blotting indicated that hydrogen-rich saline can reduce the expression levels of autophagy-related protein Beclin-1 and LC3 (Fig. 3b-c). The immunofluorescent staining showed that LC3-positive neurons were hardly observed in the hippocampus, widespread among the hippocampus after SAH induction, but they decreased after hydrogen-rich saline administration (Fig. 3d).

### Hydrogen-rich saline alleviates ER stress

**Figure 4:**
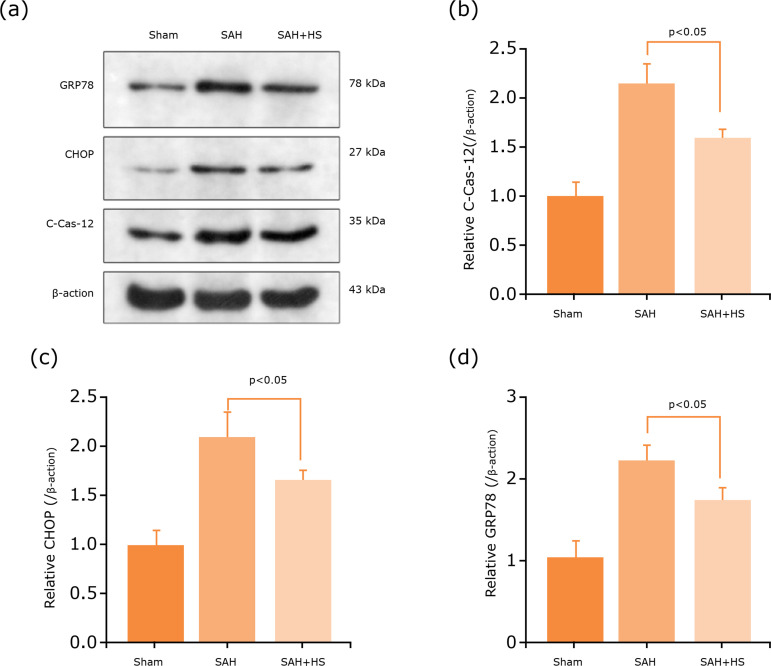
Hydrogen-rich saline alleviates ER stress. **(a)** Expression of ER stress-related proteins, caspase-12, CHOP and GRP78 in the cerebral cortex of mice after SAH were determined by Western blotting. **(b-d)** Quantification of caspase-12, CHOP and GRP78 protein levels in the cerebral cortex to actin loading control, hydrogen-rich saline decreased caspase-12, CHOP and GRP78 expression after SAH in mice. n=6; p<0.05; ANOVA; mean ± SEM.

SAH: subarachnoid hemorrhage; HS: hydrogen-rich saline; C-Cas-12: caspase-12; ER: endoplasmic reticulum stress; ANOVA: analysis of variance; SEM: scanning electron microscope.

To investigate the effect of hydrogen-rich saline on ER stress after SAH, the ER stress core markers were GRP78, CHOP, and caspase-12. We detected the expression of ER stress-associated proteins by Western blot (Fig. 4a). The results of Western blot also indicated that hydrogen-rich saline can reduce the expression levels of ER stress-related protein GRP78, CHOP, and caspase-12 (Fig. 4b-d). Hence, it is supposed that the neuroprotection of hydrogen-rich saline is partly based on ER stress inhibition.

### Rapamycin stimulates autophagy and reversed the neuroprotective effect of hydrogen-rich saline

**Figure 5:**
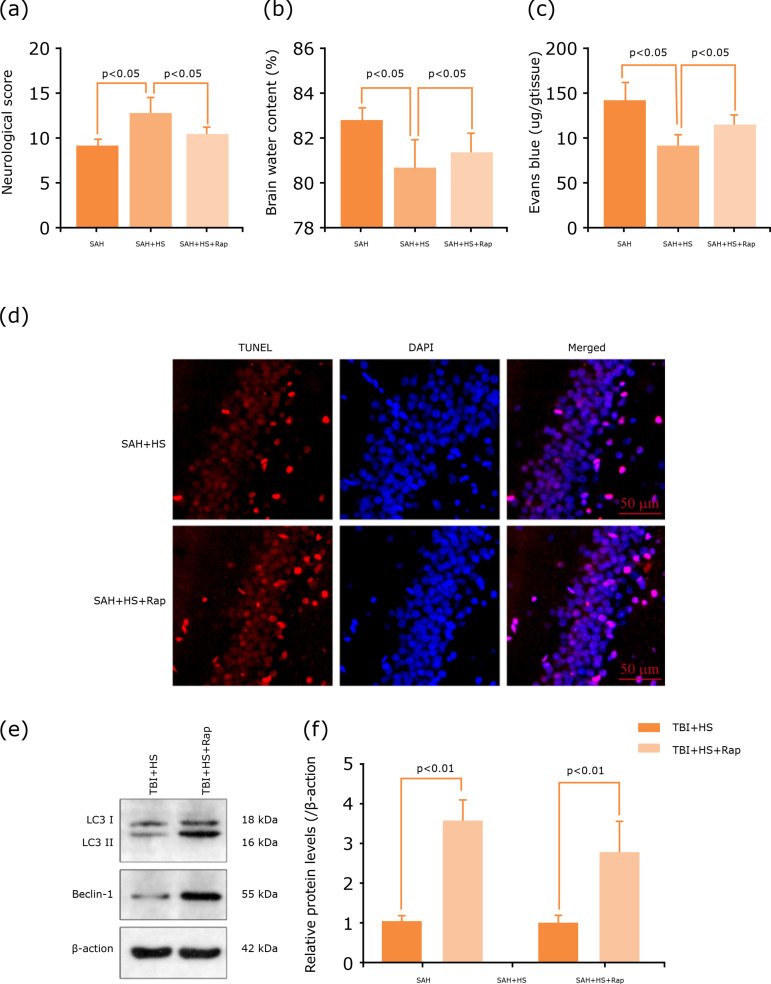
Rapamycin stimulates autophagy and reversed the neuroprotective effect of hydrogen-rich saline. **(a)** Hydrogen-rich saline increased the neurological score (n=6, p<0.05). **(b)** Hydrogen-rich saline alleviated brain water content significantly after SAH, while aggravated it after rapamycin administration (n=6, p<0.05). **(c)** Hydrogen-rich saline alleviated BBB permeability after SAH, while aggravated it after rapamycin administration (n=6, p<0.05). **(d)** TUNEL assay showed that autophagy activator increased neuronal death, and reversed the neuroprotective effect of hydrogen-rich saline. **(e)** Expression of autophagy-related proteins, LC3 and Beclin-1 after SAH were determined by Western blotting. **(f)** Rapamycin increased the expression levels of LC3 and Beclin-1 significantly than the SAH+HS group (n=6, p<0.05). n=6; p<0.05; ANOVA; mean ± SEM.

SAH: subarachnoid hemorrhage; HS: hydrogen-rich saline; Rap: rapamycin; BBB: blood-brain barrier; TUNEL: terminal deoxynucleotidyl transferase dUTP nick end labeling; ANOVA: analysis of variance; SEM: scanning electron microscope.

Rapamycin was a specific activator for autophagy^39^. To investigate the relationship between autophagy and the neuroprotective role of hydrogen-rich saline, mice were pretreated with rapamycin before the induction of SAH. The results showed that pretreated with rapamycin could dramatically damage neurological deficits (Fig. 5a), aggravate brain edema (Fig. 5b) and BBB permeability (Fig. 5c), and reverse the neuroprotective effect of hydrogen-rich saline.

Additionally, the TUNEL assay also showed that rapamycin could also significantly increase the neuron apoptosis in the injured hippocampus, compared with the SAH + hydrogen-rich saline group (Fig. 5d). The autophagy-related protein expression by Western blot was detected too (Fig. 5e). Hydrogen-rich saline can significantly decrease the expression levels of Beclin-1, and LC3 (Fig. 5f), while partly blocked with rapamycin administration. Thus, these results indicated that rapamycin could activate autophagy and abolish the anti-autophagy effects of hydrogen-rich saline, then reversed the neuroprotective effects of hydrogen-rich saline after SAH.

### Hydrogen-rich saline regulates autophagy and ER stress by ROS/Nrf2/HO-1 signaling pathway after SAH

**Figure 6:**
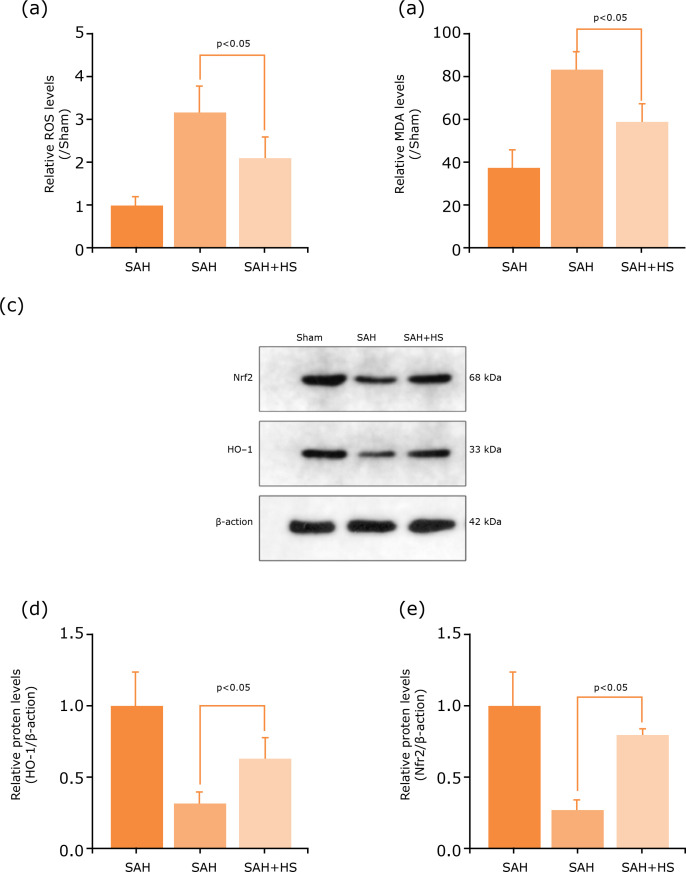
Hydrogen-rich saline regulated autophagy and ER stress by ROS/Nrf2/HO-1 signaling pathway after SAH. **(a)** Hydrogen-rich saline decreased ROS levels after SAH by DCF-DA probe. **(b)** Hydrogen-rich saline decreased MDA levels after SAH. **(c)** Expression of autophagy-related proteins, Nrf2 and HO-1 after SAH were determined by Western blotting. **(d-e)** Nrf2 and HO-1 protein levels were quantificated in the cerebral cortex to actin loading control, hydrogen-rich saline increased Nrf2 and HO-1 expression after SAH in mice. n=6; p<0.05; ANOVA; mean ± SEM.

SAH: subarachnoid hemorrhage; HS: hydrogen-rich saline; ROS: reactive oxygen species; MDA: malondialdehyde; HO-1: heme oxygenase-1; ER: endoplasmic reticulum stress; ANOVA: analysis of variance; SEM: scanning electron microscope.

Autophagy through ROS/Nrf2/HO-1 signaling pathway after hydrogen-rich treatment was explored. It was detected the ROS levels by DCF-DA probe, and the degree of membrane lipid peroxidation was evaluated by MDA. The results showed that ROS and MDA levels were significantly increased after SAH, while they decreased after hydrogen-rich treatment (Fig. 6a-b). The protein expression levels of Nrf2 and HO-1 by Western blot to investigate neuron autophagy were also detected (Fig. 6c). The results showed that the expression levels of Nrf2 and HO-1 decreased significantly in the SAH group, and increased after hydrogen-rich saline administration (Fig. 6d-e). Thus, these results showed that hydrogen-rich saline may have inhibited SAH-induced autophagy by a regulated ROS/Nrf2/HO-1 signaling pathway.

## Discussion

**Figure 7:**
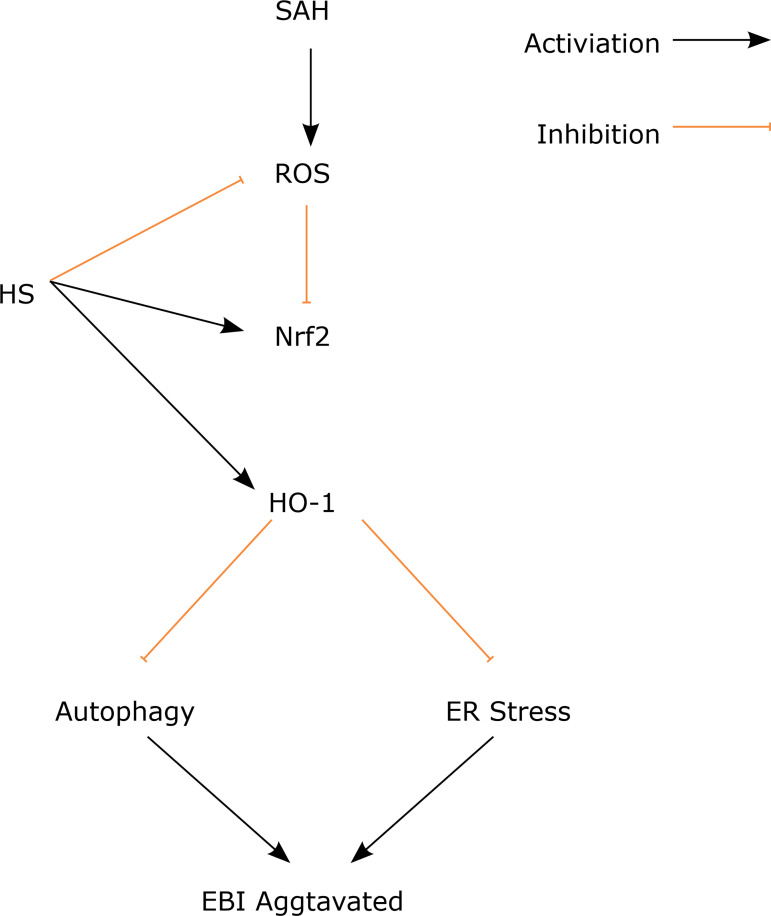
Diagram of the proposed model explaining the observations of autophagy and ER stress after SAH and potential mechanisms underlying the effect of the hydrogen-rich saline intervention.

SAH: subarachnoid hemorrhage; HS: hydrogen-rich saline; ROS: reactive oxygen species; HO-1: heme oxygenase-1; ER: endoplasmic reticulum stress; EBI: early brain injury.

Here, the therapeutic potential of hydrogen-rich saline for alleviating early brain injury in a mouse in the SAH model was evaluated. The present study demonstrates that hydrogen-rich saline was a neuroprotective agent that can attenuate EBI following SAH. It was found that hydrogen-rich saline can improve neurological dysfunction after SAH; hydrogen-rich saline can alleviate brain damage in a mouse SAH model; hydrogen-rich saline can relieve ER stress after SAH; hydrogen-rich saline can prevent autophagy after ER stress and alleviate neuronal death; and the anti-ER stress and anti-autophagy roles of hydrogen-rich saline may be related to the ROS/Nrf2/HO-1 pathway (Fig. 7).

Hydrogen gas or hydrogen-rich saline can easily penetrate the BBB by gaseous diffusion, which is widely accepted to exert protective effects in many CNS diseases, including ischemia stroke, intracranial hemorrhage, TBI, and neurodegenerative diseases^24^^-^ 27. Hydrogen gas or hydrogen-rich saline plays an important role in antioxidant activity with high tissue transferability, and previous studies had demonstrated that H_2_ is safe for patients and animals^34^. The anti-oxidative stress and anti-inflammatory response of hydrogen gas or hydrogen-rich saline are induced by selective inhibition of highly toxic ROS, such as hydroxyl radical (OH·) and peroxynitrite (ONOO−)^26^.

Liu^40^ reported that H_2_ can markedly improve the survival rate and cognitive dysfunction, decrease inflammatory response and oxidative stress, and increase activities of antioxidant enzymes in serum and hippocampus in a mouse model of sepsis. In the ICH model, it was also found that hydrogen plays a neuroprotective effect against EBI after ICH, alleviating brain edema and neurologic deficits through regulating oxidative stress, neuroinflammation, and apoptosis^41^.

In the hypoxic-ischemic brain injury neonatal rats’ model, H_2_ inhalation administration can alleviate brain damage and improve early neurological outcomes, the mechanisms also through antioxidant, antiapoptotic, and anti-inflammatory responses via MAPK/HO-1/PGC-1a pathway^42^. In the TBI model, molecular hydrogen water also can reverse the controlled cortical impact-induced brain edema through the preservation or increase of adenosine triphosphate (ATP) levels^43^. A pilot rats study indicated that high-dose hydrogen gas therapy reduces mortality and improves outcomes after SAH^44^.

Zhuang reported that hydrogen can alleviate brain injury via decreasing oxidative stress injury and brain edema in experimental SAH rabbits^32^. Hydrogen-rich saline can improve neurological function, decrease neuronal apoptosis by upregulating Bcl-2 and downregulate Bax and cleave caspase-3 after SAH. The potential mechanism may be through Akt/GSK3β signaling pathway. In the present study, we also found that hydrogen-rich saline markedly increased the survival rate and neurological score, alleviated brain edema, and increased neuron survival.

Autophagy regulates the turnover of cellular constituents to ensure the removal and recycling of toxins and was very important in cell homeostasis. The role of autophagy has been confirmed in many CNS diseases, including acute brain injury^16^^-^ 18, ICH^19^, SAH^39^, and Huntington’s disease^20^. Autophagy can transport materials in cells to lysosomes for degradation through different pathways, involved in the regulation of cell survival and death mechanisms after SAH.

Therefore, autophagy plays a very important role in neuronal injury and repair after SAH. In the myocardial ischemia/reperfusion (I/R) in vitro and in vivo model, hydrogen-rich saline can improve the inflammatory response and apoptosis via PINK1/Parkin mediated autophagy^45^. Chen^46^ reported that H_2_ can alleviate vital organ damage, inhibited lipopolysaccharide (LPS) and ATP caused by NLRP3 inflammasome pathway activation, and improve mitochondrial dysfunction via regulating autophagy. Recent studies also indicated that hydrogen-rich saline or hydrogen gas can decrease cell death via regulating autophagy^47^^-^ 50. So far, this is the first report that hydrogen-rich saline can alleviate EBI after SAH by regulating autophagy. In the present study, it was found that autophagy was excessive activated after SAH, then led to neurologic impairment, BBB disruption, brain edema, and neuronal death, while it was reversed after hydrogen-rich saline treatment.

The molecular mechanism of autophagy and ER stress is complicated, and the exact mechanisms of the neuroprotective effects of hydrogen-rich saline therapy remain unclear. Nrf2 was a very important transcriptional regulation factor that can regulate the expression of more than 250 genes and is marked by its binding site, antioxidant response element, most genes can regulate oxidative stress and cell apoptosis, necroptosis, autophagy, and ferroptosis^30^.

Yu^51^ reported that 2% molecular hydrogen (H_2_) gas inhalation can improve the survival rates, reduce the lung edema and the lung injury score, and ameliorate the injuries caused by oxidative stress and inflammation in the septic mice model. Knockout Nrf2 would reverse or weaken the protection of H_2_ gas on lung damage, and also depends on the HO-1 and high-mobility group box 1 (HMGB1).

Additionally, Chen^52^ demonstrated that H_2_ attenuates endothelial injury and inflammation, increased the HO-1 expression and in-vitro and in-vivo activity, and knockout Nrf2 or HO-1 inhibition reversed the protection of H_2_, the process depending on the activity of Nrf2/HO-1 signaling pathway. Yu^53^ reported that H_2_ can improve survival in septic mice, and decrease escape latency and platform crossing times, the brain water content, and extravascular dextran, while reversed in the Nrf2 knockout mice. Wang^42^ pointed out that hydrogen gas can alleviate hypoxic-ischemic EBI via regulating the HO-1 pathway. Intriguingly, the present study found that knockdown HO-1 reversed the neuroprotection of hydrogen-rich saline after SAH, and HO-1 might be the upstream signal of ER stress and autophagy. However, the exact mechanism needs to be further determined.

## Conclusions

The present study provided evidence that autophagy, which is mediated by the ROS/Nrf2/HO-1, is an important cellular regulatory mechanism and contributes to EBI after SAH. In this study, for the first time, it was reported that hydrogen-rich saline induced regulation of autophagy and ER stress, and also a new idea was provided to explore the biological effects and underlying mechanisms of the hydrogen-rich saline.
